# A Systematic Review on Clinical Safety and Efficacy of Vancomycin Loading Dose in Critically Ill Patients

**DOI:** 10.3390/antibiotics11030409

**Published:** 2022-03-18

**Authors:** Abdul Haseeb, Mayyasah Khalid Alqurashi, Areej Sultan Althaqafi, Jumana Majdi Alsharif, Hani Saleh Faidah, Mashael Bushyah, Amal F. Alotaibi, Mahmoud Essam Elrggal, Ahmad Jamal Mahrous, Safa S. Almarzoky Abuhussain, Najla A. Obaid, Manal Algethamy, Abdullmoin AlQarni, Asim A. Khogeer, Zikria Saleem, Muhammad Shahid Iqbal, Sami S. Ashgar, Aziz Sheikh

**Affiliations:** 1 Department of Clinical Pharmacy, College of Pharmacy, Umm Al Qura University, Makkah 24382, Saudi Arabia; m.alqurashi1999@gmail.com (M.K.A.); areejalthaqafi1@gmail.com (A.S.A.); jumana7920@gmail.com (J.M.A.); afotaibi@uqu.edu.sa (A.F.A.); merggal@uqu.edu.sa (M.E.E.); ajmahrous@uqu.edu.sa (A.J.M.); ssmarzoky@uqu.edu.sa (S.S.A.A.); 2 Department of Microbiology, Faculty of Medicine, Umm Al Qura University, Makkah 24382, Saudi Arabia; hsfaidah@uqu.edu.sa (H.S.F.); ssashgar70@hotmail.com (S.S.A.); 3 Pharmaceutical Care Services Department, King Abdullah Medical Complex, Jeddah 23816, Saudi Arabia; mbushyah@moh.gov.sa; 4 Department of Pharmaceutics, College of Pharmacy, Umm Al Qura University, Makkah 24382, Saudi Arabia; naobaid@uqu.edu.sa; 5 Department of Infection Prevention & Control Program, Alnoor Specialist Hospital, Makkah 24382, Saudi Arabia; mmalgethamy@moh.gov.sa; 6 Infectious Diseases Department, Alnoor Specialist Hospital, Makkah 24382, Saudi Arabia; al-qrni@hotmail.com; 7 Plan and Research Department, General Directorate of Health Affairs of Makkah Region, Ministry of Health, Makkah 24382, Saudi Arabia; akhogeer@moh.gov.sa; 8 Medical Genetics Unit, Maternity & Children Hospital, Makkah Healthcare Cluster, Ministry of Health, Makkah 24382, Saudi Arabia; 9 Department of Pharmacy Practice, Faculty of Pharmacy, The University of Lahore, Lahore 54000, Pakistan; xikria@gmail.com; 10 Department of Clinical Pharmacy, College of Pharmacy, Prince Sattam Bin Abdulaziz University, Alkharj 11942, Saudi Arabia; m.javed@psau.edu.sa; 11 Usher Institute, The University of Edinburgh, Teviot Place, Edinburgh EH16 4UX, UK; aziz.sheikh@ed.ec.uk

**Keywords:** vancomycin, loading dose, safety, efficacy

## Abstract

Background: The clinical significance of utilizing a vancomycin loading dose in critically ill patients remains unclear. Objective: The main aim of this systematic review is to evaluate the clinical safety and efficacy of the vancomycin loading dose in critically ill patients. Methods: We performed a systematic review using PRISMA guidelines. PubMed, the Web of Science, MEDLINE, Scopus, Google Scholar, the Saudi Digital Library and other databases were searched. Studies that reported clinical outcomes among patients receiving the vancomycin LD were considered eligible. Data for this study were collected using PubMed, the Web of Science, MEDLINE, Scopus, Google Scholar and the Saudi Digital Library using the following terms: “vancomycin”, “safety”, “efficacy” and “loading dose” combined with the Boolean operator “AND” or “OR”. Results: A total of 17 articles, including 2 RCTs, 11 retrospective cohorts and 4 other studies, met the inclusion/exclusion criteria out of a total 1189 studies. Patients had different clinical characteristics representing a heterogenous group, including patients in critical condition, with renal impairment, sepsis, MRSA infection and hospitalized patients for hemodialysis or in the emergency department. Conclusions: The study shows that the target therapeutic level is achieved more easily among patients receiving a weight-based LD as compared to patients received the usual dose without an increased risk of new-onset adverse drug reactions.

## 1. Introduction

Vancomycin, a glycopeptide antibiotic, is a type of time-dependent antimicrobial prescribed for severe infections or healthcare-associated infections caused by methicillin-resistant *Staphylococcous aureus* (MRSA) [1]. It is one of the most commonly studied antimicrobials regarding therapeutic drug monitoring in order to confirm successful clinical outcomes and to reduce the risk of nephrotoxicity [2,3,4,5]. An appropriate dosing regimen is the basis of rational vancomycin therapy in order to improve clinical outcome and reduce the development of antimicrobial resistance (AMR) and dose-dependent toxicity [6,7]. Moreover, the antimicrobial efficacy of vancomycin can be determined by the time period during which the vancomycin concentration in plasma is greater than the minimal inhibitory concentration (MIC). The antimicrobial efficacy of vancomycin is highest when the vancomycin concentration reaches 4–5 times that of the MIC [8,9]. Due to an increase in MRSA and vancomycin-resistant Enterococci infection rates, there has been an excessive use of vancomycin, which has resulted in increased MICs of more than 1.5 mg/L [10,11]. Therefore, guidelines suggest a more aggressive dose regimen of vancomycin in order achieve a target trough concentration (15–20 mg/L) for life threatening infections such as MRSA or pneumonia [12]. Recently published guidelines and literature on vancomycin therapeutic drug monitoring recommend an AUC/MIC ratio of ≥400 at MIC values of 0.5 and 1 μg/mL, comparing trough concentration targets of 10 and 20 μg/mL to optimize vancomycin exposure with minimal toxicity. Therefore, in the recent era, most clinicians prefer AUC/MIC targets over trough concentration targets to optimize vancomycin therapy [9,13].

However, most vancomycin usage in critical care settings is empiric, and the concept of AUC/MIC is pointless in these settings. An appropriately weight-based vancomycin dosing likely attains the AUC target without therapeutic drug monitoring [14]. Additionally, Gram-positive microbes cannot be efficiently eliminated if the vancomycin concentration is less than 10 mg/L. Eventually, chances of vancomycin-resistant infections increase, which may cause a prolonged hospital stay and higher mortality rate [2,15,16,17].

Dose optimization antimicrobial stewardship programs using pharmacokinetics/pharmacodynamics (Pk/Pd) principles are effective strategies to ensure clinical efficacy of most narrow therapeutic index antimicrobials [7,18]. In order to quickly achieve an effective AUC/MIC target of vancomycin and optimize its use, a loading dose (LD) of vancomycin 25–30 mg/kg (actual body weight) in adults and 20–25 mg/kg in children is recommended [19]. This practice is also supported by the revised clinical guidelines on vancomycin therapeutic drug monitoring by the IDSA [9]. Irrational dosing eventually leads to the emergence of AMR [20,21,22]. Therefore, the main reason to conduct this systematic review is to evaluate the available published data regarding the clinical safety and efficacy of the vancomycin LD in the treatment of Gram-positive infections and to provide reference for clinical practice.

## 2. Results

### 2.1. Literature Search

**Figure 1:**
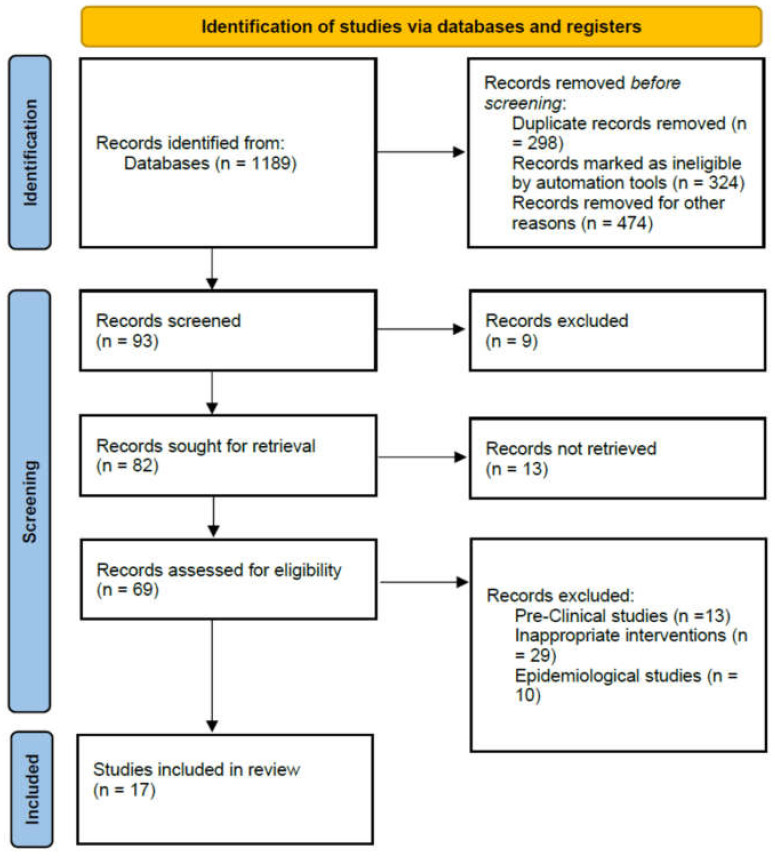
PRISMA flow diagram reporting the procedure of selection of studies.

A total of 1189 articles was identified after literature search from five databases. After applying inclusion and exclusion criteria, 93 full-text relevant articles were separated. In these research articles, nine review papers were excluded because they were not original studies. In the remaining 82 articles, 13 articles were preclinical studies and 10 epidemiology studies, 29 research papers without an LD group (LDG) and 13 research papers on unrelated topics. Finally, 17 articles, including 2 RCTs [23,24], 11 retrospective cohorts, and 4 other studies, were included [25,26,27,28,29,30,31,32,33,34,35,36,37,38,39]. The PRISMA flow diagram reporting the procedure of selection of studies is shown in Figure 1.

### 2.2. Study Characteristics

The important features of the research articles included in this review are shown in Table 1. One RCT was a double-blind study that was conducted among children aged 2–18 years old [23]. The second RCT evaluated the use of LD among patients admitted to the emergency department (ED) [24]. In the remaining 14 studies, 10 were retrospective cohort studies, 1 prospective, 1 concurrent and 2 studies conducted both retrospectively and prospectively in order to compare the results with each other. Six studies were conducted in patients with MRSA infections [25,26,27,28,29,30], while the remaining were conducted in patients with different infectious diseases [23,24,31,32,33,34,35,36,37,38,39]. The studies included intensive care unit (ICU) patients, patients with severe renal impairment, sepsis patients, hospitalized hemodialysis patients, emergency department patients and MRSA-infected patients. The quality of the included research articles was evaluated, and the results are presented in Table 2 and Table 3.

### 2.3. Attainment of Target Therapeutic Concentration and Clinical Response

Both in RCT and non-RCT subgroups, the overall attainment of target therapeutic TC (15 to 20 mg/L) was notably higher in the LDG than in the control group (CG). However, the difference was not significantly higher in the non-RCT group. The studies showed a better clinical response along with negative blood cultures in the LDG than in the CG. One observational study highlighted that the nomogram led to a reliable attainment of concentrations of vancomycin (≥15 mg/L) without increasing nephrotoxicity among patients admitted to the ICU [36]. Ortwine et al. reported that patients receiving an LD of ≥ 20 mg/kg of vancomycin on the basis of weight might not be the optimal dosing strategy [28]. Dolan and his colleagues reported that the majority of children who received an LD of 20 to 25 mg/kg of vancomycin had a subtherapeutic concentration [32].

### 2.4. Nephrotoxicity and Other Adverse Events

The total number of patients who were inflicted with renal toxicity was significantly less in the LDG than in the CG. Results of RCTs also revealed a significantly higher incidence of nephrotoxicity in the LDG. Marvin and his colleagues reported that a high LD of vancomycin does not increase nephrotoxicity when compared to a lower dose in renally impaired patients [31]. However, Demirjian et al. reported that children who received an LD of 30 mg/kg infused over 2 h reported the occurrence of nephrotoxicity and red man syndrome [23]. Besides nephrotoxicity, other common adverse drug reactions of vancomycin included flushing, pruritus and a rash. Only RCTs were compared to other adverse drug reactions between the LDG and CG, and there was no clinically significant difference between the two groups. The research articles stated the mortality rate after receiving the vancomycin LD, but there was no significant difference between the LDG and CG.

## 3. Discussion

While existing clinical practice guidelines recommend LDs of vancomycin for life threatening infections, limited published articles are available endorsing or disproving this recommendation [4,8]. Research articles evaluated in this systematic review had different methodologies, but the main data were derived from retrospective cohort studies. The recommended daily dose of vancomycin is 2 g intravenously either divided as 500 mg four times daily or 1 g twice daily for patients with a normal renal function as per directions provided by the Food and Drug Administration (FDA) of the United States. However, an FDA-approved label does not state the use of the vancomycin LD. Conversely, in various published clinical guidelines, LD is strongly recommended for patients in critically condition (including those with meningitis, sepsis, pneumonia or infective endocarditis) due to suspected MRSA infection [2,4].

Numerous research articles confirmed an improved clinical response among patients administered with an LD of vancomycin [25,30]. A meta-analysis reported that an LD group can achieve an optimal therapeutic concentration significantly better that a nonloading dose group [40]. It may take a long time for vancomycin to achieve target plasma concentrations. Therefore, in severely ill patients, an LD allows for the quick attainment of a target TC of 15–20 mg/mL. The results of RCT indicated that the vancomycin LD is a better treatment option for the management of serious infections of MRSA, compared to non-LD therapy [26]. This systematic review confirmed the results of studies stating that a LDG can attain an optimal TC significantly better than a non-LDG, whereas another study indicated that a standard dose of vancomycin (500 mg four times a day) is subtherapeutic in critically ill patients [41]. Therefore, studies suggest that a 15 mg/kg LD should be given to patients in critical condition due to suspected infections of Gram-positive microbes [42]. In most adult patients, when the LD is administered, the optimal TC (15 to 20 mg/L) can be attained within 24 h before the second dose. Likewise, the compliance rate of an optimal TC in children is higher in the LDG than in the CG [23].

Nephrotoxicity is one of the major side effects of vancomycin in patients, especially those who are critically ill [43]. Considering that the LD of vancomycin may result in a high risk of nephrotoxicity, it remains unclear whether the LD of vancomycin is safe for the treatment of serious infections. A meta-analysis reported that the risk of nephrotoxicity was lower in the LD group when compared with the control group, revealing that the LD was not associated with increased nephrotoxicity [40]. An LD of vancomycin reduces the risk of nephrotoxicity. The effective control of life-threatening infections such as sepsis through better antibacterial activity might slow down the progression of renal damage, shorten the hospitalization and reduce mortality [44]. The incidence of other adverse drug reactions (e.g., red man syndrome) was also not significantly higher in the LDG [23]. For patient safety, vancomycin should be infused slowly in the LD to avoid infusion-related adverse reactions [23,34]. Moreover, patients who receive prolonged therapy of vancomycin for more than 1 week should have their serum creatinine level checked 2–3 times weekly along with the routine monitoring of the urine output. Vancomycin antibacterial activity is time-dependent with a prolonged post antibiotic effect. Therefore, keeping the TC above an effective concentration can improve the clinical efficacy. The literature showed that a continuous vancomycin infusion along with an LD increases the clinical efficacy of vancomycin as compared to a normal infusion time [45].

Owing to the lack of a huge sample size and controlled study design, these retrospective cohorts were not enough to endorse the safety and efficacy of an LD. Controlled studies with adequate statistical analyses are required in order to endorse the clinical safety and efficacy of the LD of vancomycin. Future research should focus closely on patients with life-threatening infections, for instance, those with bacteremia, hospital-acquired pneumonia, infective endocarditis, osteomyelitis, meningitis or confirmed MRSA infections. Moreover, the development of validated vancomycin dosing nomograms should integrate well-defined guidelines related to the administration of standardized LDs in life-threatening infections to achieve a rapid TC. The use of LDs should be justified through high-quality RCTs. This systematic review had a few limitations and should be seen pragmatically. Firstly, there is a need to add more RCTs in order to validate the results, as only a few RCTs were included. Secondly, some clinical outcomes of studies included in this systematic review were not presented, as only a few studies stated the clinical outcomes, morbidity and mortality. Furthermore, heterogeneity existed in the included studies.

## 4. Materials and Methods

### 4.1. Literature Search

PubMed, the Web of Science, MEDLINE, Scopus, Google Scholar and the Saudi Digital Library were explored from their beginning up to December 2021. The search terms used for this systematic review were “vancomycin”, “safety”, “efficacy” and “loading dose” combined with the Boolean operator “AND,” or “OR”. Both text words and mesh terms were used. Additionally, references of the initially identified original research articles and related review papers were also checked for relevant research papers. Only articles written in English language were included in this systematic review.

### 4.2. Study Selection

The 2020 PRISMA guidelines (preferred reporting items for systematic reviews and meta-analyses) were used to conduct this systematic review. Randomized controlled trials (RCTs) and observational and cohort studies that presented serum TC of vancomycin after the use of vancomycin LD intravenously as either an empiric or targeted antimicrobial therapy were included. Studies that focused on laboratory research, oral vancomycin use, preclinical research, sample size of less than 10 and epidemiology were excluded. A study protocol was developed to assess eligibility for inclusion. Literature search and study selection were carried out by two independent researchers (AH and ZS). Prospective and retrospective open-label and observational studies and randomized clinical trials (RCTs) were included.

### 4.3. Data Extraction

Data extraction was carried out using the predesigned data extraction form for this systematic review. The following data were extracted from each study: (1) author(s) and year of publication; (2) reference; (3) study design; (4) sample size; (5) characteristics of the patient; (6) dosing practice; (7) outcomes that included PK data, therapeutic outcomes and toxicity; (8) inference.

### 4.4. Article Quality Assessment

The quality of each article was evaluated using a New Castle-Ottawa Scale (NOS) for retrospective studies and Cochrane bias tool for RCTs [46,47,48]. Two of the reviewers assessed the quality of each included study independently. They compared their results and disagreements were resolved by detailed discussion.

## 5. Conclusions

Multisite studies reported that the use of a vancomycin loading dose achieved optimal therapeutic and AUC/MIC targets. Moreover, the loading dose lowered the risk of nephrotoxicity and other adverse reactions. Based on the existing literature, the LD is reported to be an effective and safe treatment option for critically ill patients. However, there is a need to conduct high-quality large-scale RCTs in order to further validate the efficacy and safety of the vancomycin LD.

## Figures and Tables

**Table 1:** Loading dose of vancomycin in included studies and implications.

Author and Year	Reference	Study Design	Sample Size	Characteristics of Patients	Dosing Practice	Clinical Outcomes	Inference/Recommendation
Patients with MRSA infections							
Wesolek et al. (2018)	[25]	Single-center retrospective cohort study	124	Sepsis patients due to MRSA infection	LD: >20 mg/kg, Non-LD: <20 mg/kg	LD versus non-LD median time to SIRS resolution (h): 67 versus 109; clinical responder (improvement or culture negative): 30/37 versus 73/87	LD versus non-LD mortality: 7/37 versus 20/87; time to negative blood culture (h): 102.25 ± 71.23 versus 99.60 ± 71.06. Length of stay (h): 14.07 ± 10.03 versus 15.33 ± 8.60
Ueda et al. (2020)	[26]	Retrospective cohort	55	MRSA, MR-CoNS or Enterococcus faecium infected patients with normal kidney function	LD: of 25 mg/kg vancomycin followed by 15 mg/kg twice daily was compared with traditional dosing	When compared to usual dosage, an LD yielded early clinical results. C_min_ did not differ significantly between the regimens with and without an LD	In patients with a normal renal function, an LD of 25 mg/kg followed by 15 mg/kg twice per day did not attain the ideal Cmin at steady state
Yoon et al. (2021)	[27]	Retrospective cohorts	81	Critically ill patients with MRSA pneumonia	LDG of 25 mg/kg followed by 15–20 mg/kg every 12 h, and non-LDG	Initial LD was not linked to a better clinical outcome or rapid pharmacological target achievement	More research is needed to provide more evidence for this widely recommended practice
Ortwine et al. (2019)	[28]	Retrospective cohort	316	Patients with MRSA Bacteremia	LD ≥ 20 mg/kg and non-LD.	Initial vancomycin doses above 1750 mg were independently protective against failure without increasing the risk for nephrotoxicity	Weight-based dosing might not be the optimal strategy
Flannery et al. (2021)	[29]	Retrospective cohorts	449	Critically ill patients with MRSA infection	LD ≥20 mg/kg actual body weight and non-LD	LD was not linked to better clinical outcomes without an increased risk of AKI. Trough 10–15 mg/L: 13/469 versus 37/458 LD versus non-LD trough 15–20 mg/L: 236/469 versus 235/458. Mortality: 34/469 versus 63/458	At 12 and 24 h, LDs of 30 mg/kg versus 15 mg/kg resulted in higher trough values, but not at 36 h
Cheong et al. (2012)	[30]	Retrospective study	58	Critically ill adult patients in ICU with MRSA infections	No details provided	LD versus non-LD clinical responder (improvement or culture negative): 9/10 versus 34/48	Level II evidence
**Patients with other infections**							
Marvin et al. (2019)	[31]	Retrospective cohort	927	Severe renally impaired patients	High Ld (>20 mg/kg) vs. low dose (≤20 mg/kg) of vancomycin	LD did not increase nephrotoxicity when compared to the lower dose	Future studies on vancomycin LD should include these patients
Dolan et al. (2020)	[32]	Retrospective cohort	151	Children	LD 20 to 25 mg/kg and without a LD	More likely to attain a target TC quicker than non-LD with no significant differences in the frequency of serum creatinine or oliguria	Despite receiving vancomycin LD, the majority of children had subtherapeutic TC. A larger prospective investigation is needed to determine the impact of LD
Al-Mazraawy et al. (2021)	[33]	Retrospective cohort	223	Children	AUC24 goals were 400 to 600 mg·h/L, that incorporated trough and maximum doses	To achieve the AUC24, an increased initial dose is required. No clinical failures were detected	Only one patient had an AUC24 greater than 600 mg·h/L, and none had an AUC24 greater than 620 mg·h/L
Demirjian et al. (2013)	[23]	Single-center double- blind RCT	59	Children aged 2–18 years with different infections	LD: 30 mg/kg, infused over 2 h; non-LD: 20 mg/kg, infused over 2 h	Trough 15–20 mg/L and >20 mg/L at 8 h better attained with LD, but red man syndrome and nephrotoxicity also appeared in patients	This is level I evidence. Nephrotoxicity appeared in patients using concomitant nephrotoxins. However, the creatinine value became normal after 12 days
Rosini et al. (2015)	[24]	Single-center RCT	99	Adult ED patients with different infections	LD: 30 mg/kg (<3.6 g), MD: 15 mg/kg q12 h for three doses infused at a rate of <1000 mg/h; non-LD: 15 mg/kg (< 1–8g); MD: 15 mg/kg ql2h for three doses infused at a rate of < 1000 mg/h	LD versus non-LD; trough 15–20 mg/L at 12 h: 17/50 versus 1/49; trough 10–15 mg/L at 8 h: 23/50 versus 6/49; LD versus non-LD infusion reactions: 3/50 versus 2/49. Nephrotoxicity: 2/50 versus 3/49. Mortality: 1/50 versus 0/49	This is also level I evidence. Nephrotoxicity appeared within 24 h in a few patients. No patient needed readmission or dialysis for nephrotoxicity within 30 days
Rosini et al. (2016)	[34]	Retrospective cohort study	1330	Adult ED patients	Non-LD: >20 mg/kg; MD: not mentioned; non-LD: <20 mg/kg; MD: not mentioned	LD versus non-LD nephrotoxicity: 49/851 versus 53/479	Level II evidence
Truong et al. (2012)	[35]	Pre/postinterventionstudy	82	Adult ICU patients	LD: 2 g, infused over 4 h; MD: depend on patient clinical status; non-LD: standard therapy, MD: depend on patient clinical status	LD versus non-LD trough <15 mg/L: 18/39 versus 16/22; trough at 15–20 mg/L: 10/39 versus 4/22. Nephrotoxicity: total *n* = 4 (when trough >20 mg/L)	Level II evidence where both postintervention and preintervention groups had more nephrotoxicity
Golonia et al. (2013)	[36]	Pre/postobservational trial	117	Adult ICU patients	LD, post nomogram: 22.5–25 mg/kg (range 1000–2250 mg); non-LD: pre nomogram: standard therapy (1000 mg q12h)	LD versus non-LD trough <15 mg/L at initial pre-fourth dose: 17/60 versus 35/57. Trough 15–20 mg/L at initial pre-fourth dose *n*: 25/60 versus 11/57 trough >20 mg/L at initial pre-fourth dose *n*: 18/60 versus 11/57; LD versus non-LD nephrotoxicity: 11/60 versus 10/57	Pharmacokinetic data based on eGFR via MDRD equation and actual body weight from preimplementation group were employed to develop nomogram. Nephrotoxicity appeared after 5 days in the preimplementation and postimplementation groups
Alvarez et al. (2017)	[37]	Concurrent cohort study	41	Adult critically ill patients with sepsis	LD: 25–30 mg/kg or LD based on population pharmacokinetic parameters of the critically ill patient; MD: not mentioned; non-LD: without LD (no details); MD: not mentioned	LD versus non-LD. Trough <15 mg/L within 24 h after first dose: 7/23 versus 16/18. Trough 15–20 mg/L within 24 h after first dose: 9/23 versus 1/18. Trough >20 mg/L within 24 h after first dose: 7/23 versus 1/18.	LD versus PPK-LD versus non-LD. Scr increased: 4/11 (36.3) versus 2/12 versus 6/18; no nephrotoxicity related with vancomycin was observed
Hodiamont et al. (2021)	[38]	Prospective observational	82	Critically ill patients	LDG: 25 mg/kg; conventional dose group: 1000 mg	Achieving AUC0–24 ≥ 400 mg·h/L was more significant in patients who received a weight-based LD of 25 mg/kg, without increased the risk of new-onset AKI	Patients with AUC0–24 > 400 mg·h/L had a considerably greater risk of AKI
Denetclaw et al. (2013)	[39]	Retrospective observational trial	69	Adult ICU patient	Initial dose: two doses of 15 mg/kg	Average TC (mg/L): 15.1 ± 3.4 and TC ≥14.8 mg/L by second dose	Initial TC not significantly different in patients with severe sepsis vs. not severe sepsis

AKI: acute kidney injury; AUC: area under curve; EF: Enterococcus faecium; ICU: intensive care unit; LD: loading dose; LDG: loading dose group; MD: median dose; MRSA: methicillin-resistant *Staphylococcus aureus*; MR-CoNS: methicillin-resistant coagulase-negative Staphylococci; RCT: randomized control trial; TC: trough concentration.

**Table 2:** Quality assessment of cohort studies.

Author and Year	Reference	Representation of Exposed Cohort	Selection of Non exposed Cohort	Ascertainment of Exposure	Demonstration that Outcome of Interest Was not Present at Start of Study	Comparability of Cohorts on the Basis of the Design or Analysis	Assessment of Outcome	Adequacy of Follow Up of Cohorts	Score
Hodiamont et al. (2021)	[38]	Yes	Yes	Yes	Yes	Yes	Yes	Yes	7
Ueda et al. (2020)	[26]	Yes	Yes	Yes	Yes	Yes	Yes	Yes	8
Yoon et al. (2021)	[27]	Yes	Yes	Yes	Yes	Yes	Yes	Yes	8
Ortwine et al. (2019)	[28]	Yes	Yes	Yes	Yes	Yes	Yes	Yes	7
Flannery et al. (2021)	[29]	Yes	Yes	Yes	Yes	Yes	Yes	No	6
Marvin et al. (2019)	[31]	Yes	Yes	Yes	Yes	Yes	Yes	No	7
Dolan et al. (2020)	[32]	Yes	Yes	Yes	Yes	Yes	Yes	No	6
Al-Mazraawy et al. (2021)	[33]	Yes	Yes	Yes	Yes	Yes	Yes	No	7
Cheong et al. (2012)	[30]	Yes	Yes	Yes	Yes	Yes	No	No	6
Truong et al. (2012)	[35]	Yes	Yes	Yes	Yes	Yes	Yes	Yes	8
Golonia et al. (2013)	[36]	Yes	Yes	Yes	Yes	Yes	Yes	No	7
Rosini et al. (2016)	[34]	Yes	Yes	Yes	Yes	Yes	Yes	No	7
Alvarez et al. (2017)	[37]	Yes	Yes	Yes	Yes	Yes	Yes	Yes	8
Wesolek et al. (2018)	[25]	Yes	Yes	Yes	Yes	Yes	No	Yes	8
Denetclaw et al. (2013)	[39]	Yes	Yes	Yes	Yes	No	Yes	Yes	7

**Table 3:** Risk of bias assessment for randomized controlled trials.

Study	References	Random Sequence Generation	Allocation Concealment	Blinding of Participants and Personnel	Blinding of Outcome Assessment	Incomplete Outcome Data	Selective Reporting	Other Bias
Demirjian et al. (2013)	[23]	Low risk	Low risk	Low risk	Low risk	Low risk	Low risk	Unclear
Rosini et al. (2015)	[24]	Low risk	Low risk	Low risk	Low risk	Low risk	Low risk	Unclear

## Data Availability

Not Applicable.
